# Desmoglein 2 Is Less Important than Desmoglein 3 for Keratinocyte Cohesion

**DOI:** 10.1371/journal.pone.0053739

**Published:** 2013-01-11

**Authors:** Eva Hartlieb, Bettina Kempf, Miriam Partilla, Balázs Vigh, Volker Spindler, Jens Waschke

**Affiliations:** 1 Institute of Anatomy and Cell Biology, Ludwig-Maximilians University Munich, Munich, Germany; 2 Institute of Anatomy and Cell Biology, Julius-Maximilians University, Würzburg, Germany; Northwestern University Feinberg School of Medicine, United States of America

## Abstract

Desmosomes provide intercellular adhesive strength required for integrity of epithelial and some non-epithelial tissues. Within the epidermis, the cadherin-type adhesion molecules desmoglein (Dsg) 1–4 and desmocollin (Dsc) 1–3 build the adhesive core of desmosomes. In keratinocytes, several isoforms of these proteins are co-expressed. However, the contribution of specific isoforms to overall cell cohesion is unclear. Therefore, in this study we investigated the roles of Dsg2 and Dsg3, the latter of which is known to be essential for keratinocyte adhesion based on its autoantibody-induced loss of function in the autoimmune blistering skin disease pemphigus vulgaris (PV). The pathogenic PV antibody AK23, targeting the Dsg3 adhesive domain, led to profound loss of cell cohesion in human keratinocytes as revealed by the dispase-based dissociation assays. In contrast, an antibody against Dsg2 had no effect on cell cohesion although the Dsg2 antibody was demonstrated to interfere with Dsg2 transinteraction by single molecule atomic force microscopy and was effective to reduce cell cohesion in intestinal epithelial Caco-2 cells which express Dsg2 as the only Dsg isoform. To substantiate these findings, siRNA-mediated silencing of Dsg2 or Dsg3 was performed in keratinocytes. In contrast to Dsg3-depleted cells, Dsg2 knockdown reduced cell cohesion only under conditions of increased shear. These experiments indicate that specific desmosomal cadherins contribute differently to keratinocyte cohesion and that Dsg2 compared to Dsg3 is less important in this context.

## Introduction

Desmosomes facilitate intercellular adhesive strength in epithelial and some non-epithelial tissues. Desmogleins (Dsg) and desmocollins (Dsc) build the core of desmosomes [1], [2]. Dsg and Dsc are Ca^2+^-dependent adhesion proteins of the cadherin family which are, beside their localization in desmosomes, also present on the cell membrane outside of desmosomes [3]. Cell cohesion is provided by transinteraction of the extracellular N-terminal domain of specific desmosomal cadherin isoforms from adjacent cells. The C-terminal end spans the plasma membrane and binds to the armadillo proteins plakoglobin and plakophilin which are anchored to the keratin filament cytoskeleton via desmoplakin. In the epidermis, a total of four Dsg (Dsg1-4) and three Dsc (Dsc1-3) isoforms are expressed [1], [2]. Recently it was shown by extracellular crosslinking experiments that Dsg2 similar to Dsc2, Dsg3 and Dsc3 is engaged in homophilic trans-interaction on the keratinocyte cell surface [4]. However, the contribution of the specific isoforms to overall cell cohesion has not been determined so far. Dsg3 has been identified as one of the autoantigens in the autoimmune blistering skin disease pemphigus vulgaris (PV) [5]. In this disease, circulating autoantibodies targeting Dsg1 and Dsg3 induce loss of cell cohesion (termed acantholysis) within the epidermis and mucous membranes. The expression of Dsg3 is mainly restricted to stratified epithelia. In the epidermis, it is expressed throughout the basal and the spinous layer [1], [2]. In contrast, Dsg2 is the most widespread desmoglein isoform. It is most abundant in the myocardium and in simple epithelia such as the intestinal mucosa [6], [7], and has been demonstrated to be expressed in the hair follicle and also in the basal epidermal layer [1], [2], [8]. In intestinal epithelial cells, Dsg2 contributes to monolayer integrity and epithelial barrier function because a monoclonal inhibitory antibody targeting the Dsg2 extracellular domain caused loss of cell cohesion and transepithelial resistance and furthermore disturbed tight junction morphology [9]. However, the specific function of Dsg2 in the epidermis and its role for maintenance of tissue integrity is largely unknown. Recently, a novel role for Dsg2 as a binding partner for caveolin-1 has been reported [10]. Via this interaction Dsg2 might be involved in desmosome turnover and intracellular signaling events. The aim of this study was to clarify the role of Dsg2 for cell cohesion in keratinocytes. We provide evidence that Dsg2, when compared to Dsg3, is less important for cell-cell adhesion but is required for keratinocyte cohesion under conditions of increased mechanical stress indicating that the contribution of specific desmosomal cadherin isoforms to overall adhesive strength and tissue integrity is different.

## Materials and Methods

### Antibodies and Reagents

Following primary antibodies were used to detect proteins by immunostaining and/or Western blot analysis: anti-Dsg1 (clone P124, Progen, Heidelberg, Germany), anti-Dsg2 mAb (clone 10G11, Progen, custom-made without any preservation components), anti-Dsg3 pAb (clone H-145, Santa Cruz Biotechnology, Santa Cruz, California), anti-Dsg3 mAb (clone 5G11, Life Technologies, Carlsbad, California), anti-Dsc1 pAb (clone L-15, Santa Cruz Biotechnology), anti-Dsc2 pAb (Progen), anti-Dsc3 mAb (clone U114, Progen), anti-ß-Actin mAb (Sigma, St.Louis, USA), anti-E-Cadherin mAb (clone 36, BD Biosciences), anti-Desmoplakin mAb (Epitomics, California, USA), anti-α-Tubulin mAb (Abcam, Cambridge, UK). HRP-linked anti-rabbit IgG Ab (Cell Signaling), anti-mouse IgG+IgM Ab and anti-goat IgG (Dianova, Hamburg, Germany) and Cy2- or Cy3-labeled goat-anti mouse, goat-anti rabbit and donkey-anti goat antibodies (Dianova) were used as secondary antibodies. Alexa Fluor®488 phalloidin (Life Technologies, Eugene, USA) was used to stain the actin filaments and was applied together with the secondary antibodies. The monoclonal Dsg2 Ab (Progen, without NaN_3_; host: mouse, isotype: IgG1) and AK23 (host: mouse, isotype: IgG), a monoclonal pathogenic antibody derived from a pemphigus mouse model [10], were utilized for the incubation steps in the cell culture model.

### Cell Culture

The spontaneously immortalized human keratinocyte cell line HaCaT [11], the colorectal adenocarcinoma HT-29 and the Caco-2 cell line purchased from American Type Culture Collection (ATCC, Manassas, USA) were grown in Dulbeccós modified Eagle Medium (DMEM, Life Technologies) supplemented with 10% fetal bovine serum (Biochrom, Berlin, Germany), 50 U/ml penicillin (AppliChem, Darmstadt, Germany) and 50 µg/ml streptomycin (AppliChem) and maintained in a humidified atmosphere containing 5% CO_2_ at 37°C. For culturing the squamous cell carcinoma cell line SCC9, a 1∶1 mixture of DMEM and F-12 nutrient mixture (Ham) (Life Technologies) supplemented with 10% fetal bovine serum, 50 U/ml penicillin and 50 µg/ml streptomycin was used. For all experiments, HaCaT cells were grown to full confluence within 4 days in high calcium medium (1.8 mmol/l CaCl_2_) to ensure that the keratinocytes express both Dsg2 and Dsg3 and were treated at a similar differentiation stage. For the dispase-based dissociation assay, Caco-2 cells were cultured for 14 days, HT-29 cells for 4 days and SCC9 cells for 5 days. These time points were selected based on the characterization of the expression profile of Dsg2 in the respective cell line.

### siRNA-mediated Knockdown

ON-TARGET plus SMARTpool siRNA for human Dsg2 (L-011645-00-0005) or Dsg3 (L-011646-00-0005) and non-targeting control siRNA (D-001810-10-05) were purchased from Thermo Scientific/Dharmacon (Lafayette, Colorado, USA). For transfection, HaCaT cells were cultured to 70–80% confluence in 24-well plates within 24 hours. By using the in vitro transfection reagent TurboFect™ (Fermentas, Thermo Scientific, Waltham, Massachusetts, USA), according to the manufactures protocol, cells were incubated with a final concentration of 0.6 µg siRNA for 24 hours. For each experimental setup, cells in the same multiwell plate were transfected with non-targeting siRNA as a control and knockdown efficiency was always proven by Western blot analysis or immunostaining.

### Ca^2+^-switch Assay

HaCaT cells were cultured to confluence on glass coverslips within 4 days. To eliminate Ca^2+^ from the medium EDTA was added for 1 hour at a final EDTA concentration of 5 mM (Ca^2+^-depletion). Cells were washed twice with medium to remove any remaining EDTA and were then incubated for further 18 hours with high-Ca^2+^ medium (Ca^2+^-repletion) to allow cell-cell contact formation. One set of coverslips was additionally incubated with either Dsg2 mAb or AK23 (each at a concentration of 2.5 µg/ml) to test whether the antibodies block cell-cell-contact formation and redistribution of Dsg2 or Dsg3 on the cell membrane. HaCaT cells without any treatment and cells without Ca^2+^-repletion were used as a control.

### Immunostaining

Cells grown on glass coverslips were fixed for 10 minutes with 2% formalin (freshly prepared from paraformaldehyde) in PBS at room temperature and then permeabilized with 0.1% Triton X-100 in PBS for 5 minutes. After blocking with 3% bovine serum albumin and 1% normal goat serum for 40 minutes cells were incubated with the respective primary antibodies over night at 4°C. Then appropriate secondary antibodies were incubated for 1 hour at room temperature and the glass coverslips were finally mounted with n-propyl gallate as antifading compound. Intact human epidermis samples were embedded immediately in tissue freezing medium (Leica Microysystems, Nussloch, Germany) and stored at −80°C. After 30 min heating to 60°C and 1 h incubation with 1% Triton X-100 in PBS, immunostaining of skin cryosections was performed as described for cell culture monolayers. Images of immunostained samples were acquired using a Leica SP5 confocal microscope with a 63× NA 1.4 PL APO objective (both Leica Microsystems, Wetzlar, Germany).

### Western Blot Analysis

For western blot analysis, cells were cultured in 24-well plates for 4 days. After washing with PBS, cells were scraped in SDS-lysis buffer containing 25 mmol/l HEPES, 2 mmol EDTA, 25 mmol/l NaF and 1% sodiumdodecylsulfate (pH 7.4). Lysates were immediately sonicated, boiled at 95°C for 2 minutes and stored at −80°C. The BCA protein assay kit (Pierce/Thermo Scientific) was used to determine the protein concentration according to the manufacturer’s protocol. Prior to separation of the proteins on polyacrylamide gels, samples were mixed with Laemmli buffer [11]. After transferring the proteins to nitrocellulose membranes and a following blocking step with 5% milk solution, primary antibodies were incubated over night at 4°C. Adequate horse-radish peroxidase conjugated secondary antibodies were incubated for 2 hours at room temperature and an ECL reaction (self-made solutions) was used to visualize the proteins.

### Triton X-100 Protein Fractionation

Protein fractionation was carried out as described previously [12]. To divide cell lysates into Triton-soluble and -insoluble fractions, HaCaT cells were incubated with Triton-buffer containing 0.5% Triton X-100, 50 mmol/l MES, 25 mmol/l EGTA and 5 mmol/l MgCl_2_ for 15 minutes on ice under gentle shaking. By centrifugation at 13000 rpm for 5 minutes cell lysates can be divided into the Triton- soluble (supernatant) and -insoluble (pellet) fraction. After measuring the protein concentration as described above both fractions were mixed with Laemmli buffer and subjected to Western blotting.

### Dispase-based Dissociation Assay

The dissociation assay was performed as described previously [13] with some modifications. Depending on the experimental setup, cells were either incubated with monoclonal antibodies for indicated times or transfected with siRNA in 24-well plates. The confluent cell monolayers were washed with Hanks buffered saline solution (Hbss) and released from the well bottoms by a 20 min incubation step with 150 µl of the enzyme dispase (>2.4 U/ml Dispase II in Hbss, Sigma, St. Louis, USA) at 37°C. After addition of 200 µl Hbss, cell monolayers were exposed to mechanical stress by pipetting them five times with a 1 ml electrical pipette. For experiments applying higher mechanical shear monolayers were pipetted 12 times. A binocular stereo microscope (Leica, Mannheim, Germany) was used to count the resulting fragments.

### Laser Tweezers Experiments

Recombinant Dsg2-Fc or Dsg3-Fc proteins were purified from cell culture supernatants of stably transfected Chinese ovarian hamster (CHO) cells by protein A agarose (Calbiochem/Merck, Darmstadt, Germany) columns as described elsewhere [14]. For laser tweezers experiments, protein A-coated microbeads (2,4 µm diameter, Life Technologies) were coated with recombinant Dsg2 or Dsg3 protein consisting of the extracellular domain of either Dsg2 or Dsg3 and the Fc part of human IgG1. These beads were then allowed to settle on the surface of HaCaT cells grown on coverslips for 30 minutes in DMEM. The binding of 100 Dsg-coated microbeads was assessed by a home-built laser tweezers setup [15]. The number of beads that could not be displaced by the beam of a 1,064 nm Nd:Yag laser (42 mW in the focal plane) is a measure for Dsg2- or Dsg3-mediated binding.

### Atomic Force Microscopy

An atomic force microscopy (AFM) setup (Bioscope AFM driven by a Nanoscope III controller, both Bruker Nano, Karlsruhe, Germany) was used to assess the capability of Dsg2 mAb and AK23 to interfere with homophilic Dsg2 and Dsg3 binding as described in detail elsewhere [16]. Briefly, freshly cleaved mica sheets (SPI supplies, Unterfoehring, Germany) and Si_3_N_4_ AFM cantilevers (Bruker Nano, Mannheim, Germany) were functionalized with Dsg3-Fc or Dsg2-Fc respectively. Cantilevers were then brought in contact with the mica to allow for binding of Dsg molecules on the cantilever tip and on the mica sheet and then retracted again. During these trace-retrace cycles, the deflection of the cantilever is monitored. In case of a binding event, the cantilever will be bent downwards towards the mica sheet during the retraction phase due to the adhering Dsg molecules. When the pulling force exceeds the adhesive forces between the Dsg molecules, the bond ruptures and the bent cantilever jumps back into its neutral position. The numbers of these so called unbinding events per total number of force-retrace cycles (binding frequency) were counted and compared before and after antibody treatment. Parameters were applied as follows: Pulling length (ramp size): 300 nm; pulling speed: 600 nm/s; cantilever contact time with the mica sheet: 100 ms. At least 2 different cantilever/mica combinations with roughly 400 trace-retrace cycles per cantilever/mica combination were used for every condition.

### Statistics

Band densitometries of Western blot experiments were quantified by ImageJ software. Error bars in graphs represent standard error of mean (SEM). Statistical significance was calculated either by Students t-test or by Mann Whitney test depending on Gaussian distribution of two-group samples and by ANOVA followed by Bonferroni’s correction for multiple-group samples. Significance was implied for p<0.05.

## Results

### Both Dsg2 and Dsg3 are Expressed in Confluent Human Keratinocytes (HaCaT)

**Figure 1:**
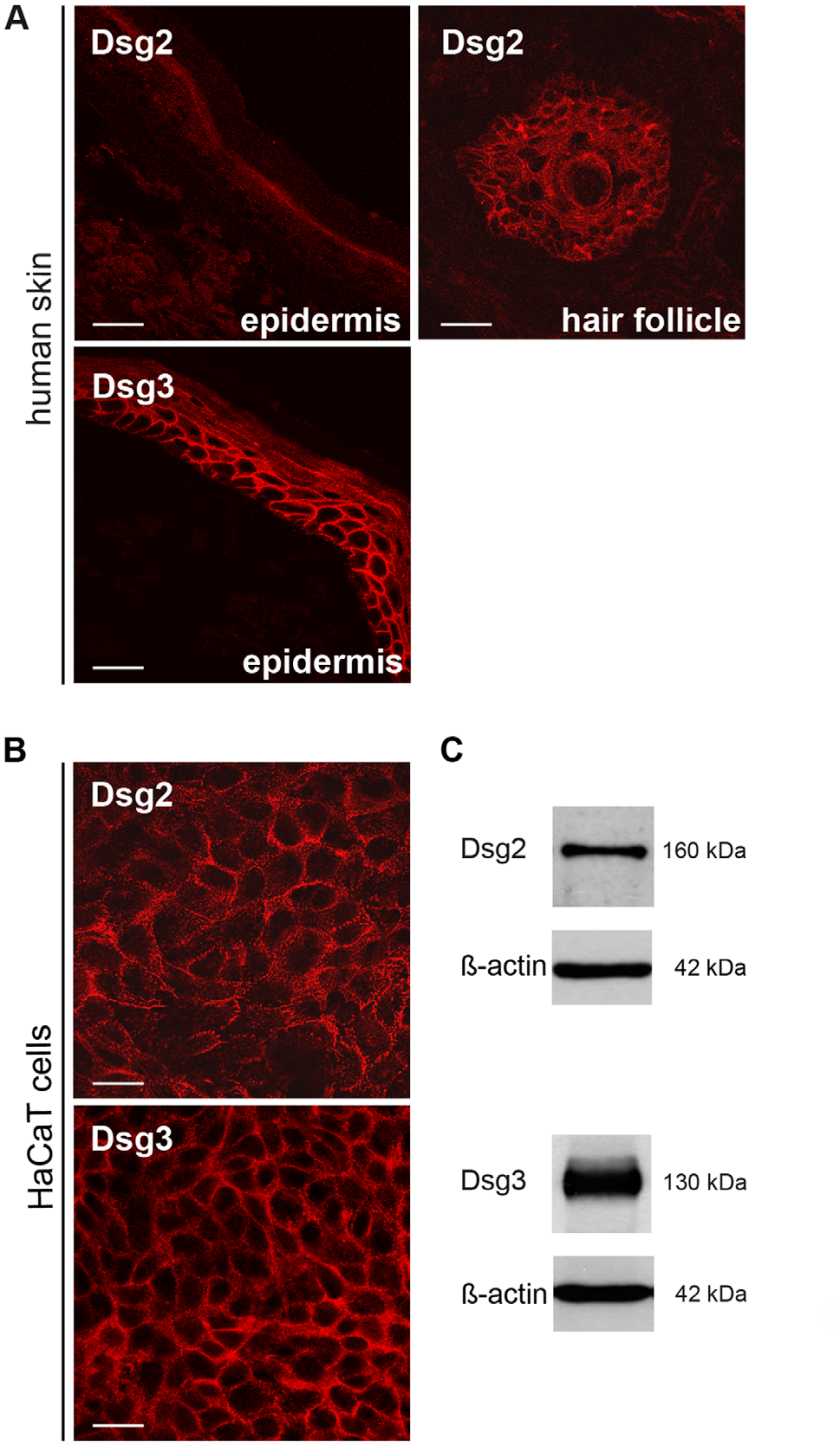
Expression patterns of Dsg2 and Dsg3 in human keratinocytes. (A) Immunofluorescence staining of cryosections of human skin demonstrating expression of Desmoglein (Dsg) 2 and Dsg3 in epidermis. For Dsg2 also the expression in the hair follicle is shown. Scale bar, 20 µm. (B) Immunofluorescence staining of Dsg2 and Dsg3 in a human keratinocyte cell line (HaCaT). Cells were cultured for 4 d in high Ca^2+^-medium. Scale bar, 20 µm. (C) Western blots analysis of Dsg2 and Dsg3 in confluent 4 d HaCaT cells. ß-actin was used as loading control.

Desmosomal cadherins show a layer-specific expression pattern in human epidermis. Dsg2 was reported to be expressed in the hair follicle [17] whereas Dsg3 was also detected throughout basal and spinous epidermal layers [1], [2], [8]. We evaluated this distribution pattern in intact human skin and confirmed that Dsg2 was strongly expressed in the external root sheath of the hair follicle whereas Dsg3 was found throughout the epidermis except the granular layer. However, Dsg2 was not detectable in human adult epidermis outside of hair follicles which was reported similarly by others [18], [19] (Fig. 1 A).

In our cell culture model (HaCaT) we determined the conditions when both Dsg2 and Dsg3 were expressed. Confluent HaCaT cells grown in Dulbeccós modified eagle medium containing 1.8 mmol/l Ca^2+^ were analyzed for the expression of Dsg2 and Dsg3. After 4 days in culture the cells formed a fully confluent monolayer. Both proteins were detectable by immunostaining in typical localization along the cell membrane (Fig. 1 B). Dsg2 appeared to be present in a more punctate pattern whereas Dsg3 staining revealed a more continuous distribution along the cell membrane. Protein expression of both Dsg isoforms under the conditions used for this study was confirmed by Western blot analysis of total HaCaT cell lysates (Fig. 1 C). Similarly, Dsc2 and Dsc3 were detectable in HaCaT cells by immunfluorescence and Western blot analysis, whereas Dsg1 and Dsc1 were not present on the cell membrane (Fig. S1 A and S2 B). However after 8d of culture, Dsg1 was present in HaCaT cells (Fig. S1 B). Since Dsg4 has been reported to be expressed in highly differentiated keratinocytes cultured for more than 6 days only [20], expression of Dsg4 was not further characterized here.

### Antibody-mediated Targeting of Dsg3 but not of Dsg2 Interaction Induces Cell Dissociation in Human Keratinocytes

**Figure 2:**
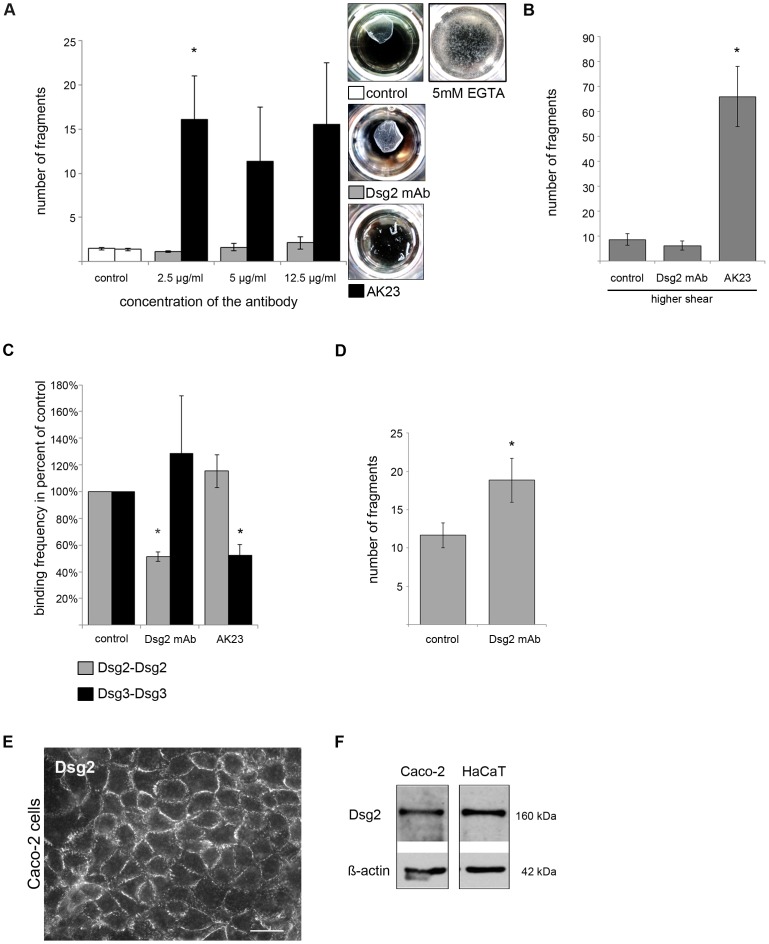
Antibody-mediated targeting of Dsg3, but not of Dsg2, led to profound loss of cell cohesion. (A) After antibody incubation for 24 h with either a monoclonal Dsg2 antibody (Dsg2 mAb) or AK23, confluent HaCaT monolayers were subjected to dispase-based dissociation assays. Loss of cell cohesion was detectable in cells incubated with AK23 only. (n = 8; * p<0.05 vs. control) Photos were taken immediately after assay performance. 1 hour incubation with 5 mM EGTA led to profound loss of cell cohesion indicating the non-hyperadhesive state of HaCaT cells used for these experiments. (n = 6) (B) Exposing the cells to more severe mechanical stress in dissociation assays increased fragment numbers after AK23 treatment only. (n = 6; * p<0.05 vs. control) (C) Atomic force microscopy was used to demonstrate antibody-mediated interference with homophilic Dsg2 and Dsg3 binding. Both antibodies reduced the binding frequency of their respective antigens. (>1000 force distance cycles on more than 2 different cantilever/substrate combinations; * p<0.05 vs. control) (D) Dispase-based dissociation assay performed with confluent Caco-2 cells after 24 h incubation with Dsg2 mAb showed a significant increase in fragment numbers compared to control cells. (n≥20; * p<0.05 vs. control) (E) Protein expression of Dsg2 in Caco-2 cells was proven by immunofluorescence (scale bar, 20 µm) and (F) Western blot analysis. ß-actin was used as loading control.

Binding of desmosomal cadherins can be blocked by specific autoantibodies against the extracellular domains (EC1-5) [16,21,22,23]. Here, we applied a monoclonal Dsg2 antibody (mAb) targeting the extracellular domain which we have shown to be inhibitory in a previous study [9] as well as AK23, an inhibitory monoclonal antibody from an active pemphigus mouse model targeting the Dsg3 adhesive interface [21], [22]. We tested these mAbs in the dispase-based dissociation assay which provides a useful tool to determine cell-cell adhesion in confluent HaCaT monolayers [22]. Cells were incubated with different concentrations of either Dsg2 mAb or AK23. After 24 h incubation, cell monolayers were released from the well bottoms with dispase, exposed to mechanical stress, and resulting fragments were counted. Under control conditions, monolayers were largely capable to withstand mechanical stress (fragment number: 1.40±0.11) (Fig. 2 A). First, we evaluated whether HaCaT cells under our experimental conditions displayed Ca^2+^-dependent binding. Because incubation with 5 mM EGTA for 1 h resulted in numerous cell fragments, cell cohesion of HaCaT cells obviously was Ca^2+^-dependent and thus had not acquired a hyperadhesive state [24], [25] (Fig. 2 A). After incubation with up to 12.5 µg/ml Dsg2 mAb, only a minor increase in fragment numbers (2.13±0.69) was detectable whereas incubation with 2.5 µg/ml of AK23 led to a significant loss of cell cohesion (16.09±4.92) (Fig. 2 A). Exposing cell monolayers to more intensive mechanical stress resulted in increased fragment numbers after AK23 incubation (66.00±12.00) whereas cells incubated with Dsg2 mAb showed again similar fragment numbers (6.17±1.85) as under control conditions (8.67±2.25) (Fig. 2 B). To rule out that the observed effects were cell-type specific, we incubated SCC9 cells which had a similar expression profile of desmosomal cadherins like HaCaT cells except that they expressed Dsg1 with both antibodies (Fig. S2 A and B). Nevertheless, no membrane staining for Dsg1 was detectable in SCC9 cells (data not shown). Again AK23 led to loss of cell-cell cohesion whereas Dsg2 mAb had no effect (Fig. S2 C). To confirm that both antibodies are potent to inhibit Dsg2 and Dsg3 interaction, we quantified binding events of recombinant Dsg molecules in a cell-free system using atomic force microscopy (AFM). Similar to previous studies, both mAbs led to a decrease in the number of binding events (binding frequency) of their corresponding target protein compared to untreated controls [9], [22], [23]. Dsg2 mAb reduced homophilic Dsg2 interaction to 51.3±3.7% of control, but not homophilic Dsg3 interaction (128.8±42.9% of control). Vice versa, AK23 decreased homophilic Dsg3 binding frequency to 52.1±8.4% of controls but not that of homophilic Dsg2 (115.4±12.2% of control) (Fig. 2 C). We then checked whether antibodies interfered with formation of cell contacts. However, in a Ca^2+^-switch assay neither the Dsg2 mAb nor AK23 were capable to interfere with reconstitution of Dsg2- and Dsg3-containing cell contacts (Fig. S3). Binding of both Dsg2 mAb and AK23 to Dsg2 or Dsg3 within desmosomes was proven by detection of both antibodies’ heavy chains in the Triton X-100-insoluble fractions (Fig. S4). To further substantiate the functionality of the Dsg2 mAb, we performed dispase-based dissociation assays in a colon carcinoma cell line (Caco-2), in which Dsg2 is the only desmosomal cadherin expressed beside Dsc2 [7], [26] (Fig. 2 E and F). Similarly as shown previously [9], 24 h incubation of Caco-2 cells cultured for 14 days in high-calcium with Dsg2 mAb led to a significant increase in fragment numbers to 18.85 (±2.87) compared to control conditions with an average fragment number of 11.65 (±1.65) (Fig. 2 D). Dsg2 was also found to be important in another colorectal adenocarcinoma cell line, HT-29. SiRNA-mediated depletion of Dsg2 resulted a significant increase in fragment numbers (12.3±0.67) in the dispase-based dissociation assay compared to cells transfected with non-targeting siRNA (3.87±0.26) (Fig. S2 D and E). Taken together, Dsg2 mAb in contrast to AK23 did not reduce cell-cell adhesion in a human keratinocyte cell line as well as the squamous carcinoma cell line SCC9, whereas it was readily impairing monolayer integrity in cells expressing Dsg2 as the only Dsg isoform.

### Loss of Both Dsg3 Binding and Cell Cohesion is Independent of Autoantibody-induced Dsg3 Depletion

**Figure 3:**
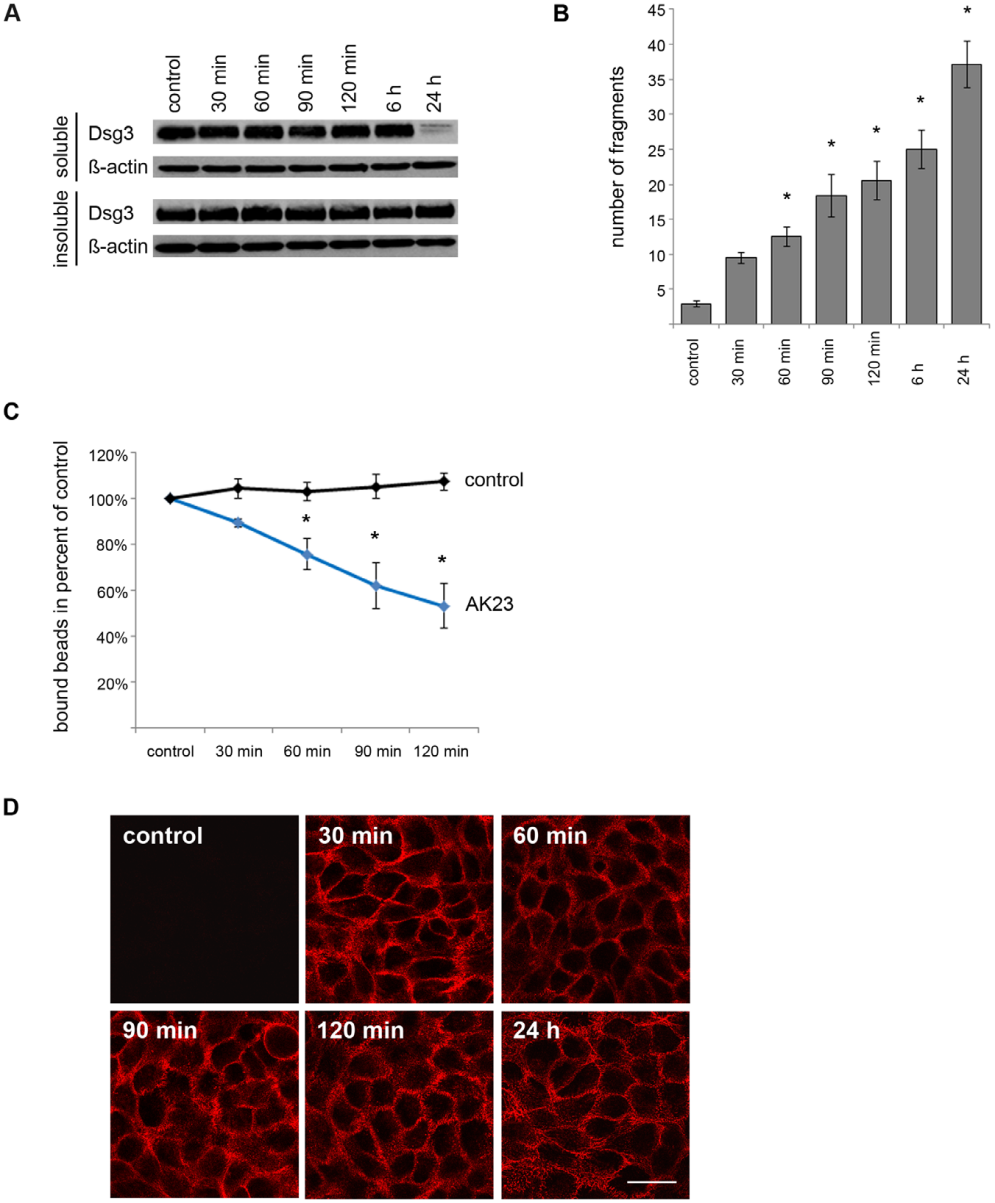
Depletion of Dsg3 is an event subsequent to loss of cell-cell adhesion after AK23 incubation. (A) 24 hours after incubation with AK23 depletion of Dsg3 was detectable in the Triton-soluble but not in the Triton-insoluble fraction of HaCaT cell lysates. ß-actin was used as loading control. (n = 3) (B) Loss of cell cohesion started after 30 min of AK23 incubation as detected in the dispase-based dissociation assays. (n = 6; * p<0.05 vs. control) (C) Similarly, laser tweezers experiments to evaluate binding of Dsg3-coated microbeads on HaCaT cell surface showed a reduction of tightly attached beads starting after 30 min following AK23 exposure. (n = 6; * p<0.05 vs. control) (D) AK23 binding to the cell surface at all time points of laser tweezers measurements was demonstrated by immunostaining for mouse Fc after fixation. Scale bar, 20 µm.

A series of time course experiments was performed *in vitro* to test whether autoantibody-induced loss of cell adhesion is caused by Dsg3 depletion or occurs as a depletion-independent event. AK23 was applied to confluent HaCaT cells at different time points within a range between 30 minutes and 24 h. By incubation with a Triton X-100 containing buffer and subsequent centrifugation the HaCaT cell lysates were divided into an extradesmosomal (Triton X-100-soluble) and a desmosome-containing fraction (Triton X-100-insoluble). In Western blot analysis, reduced levels of Dsg3 were detectable in the Triton-soluble fraction after 24 h incubation time only. The amount of Dsg3 in the Triton-insoluble fraction remained unchanged during the complete time course (Fig. 3 A). In contrast, loss of cell adhesion as detected by the dispase-based dissociation assay appeared as an initial but time-dependent event and was detectable as soon as after 30 minute incubation with AK23, at a time point in which no depletion of Dsg3 was detectable (Fig. 3 B). Consistent with these findings, microbeads coated with recombinant Dsg3 showed a less pronounced binding capacity to the surface of HaCaT cells when AK23 was incubated for 30 minutes (89.48% ±1.75% bound beads). The number of bound microbeads was even more reduced when AK23 was applied for longer time periods with maximum reduction after 120 minutes (53.27% ±9.89% bound beads). Under control conditions no changes in the number of bound beads was detectable (Fig. 3 C). At all time points used in the laser tweezers experiments, AK23 was clearly detectable at cell-cell contacts by a goat-anti mouse Cy3-labeled secondary antibody (Fig. 3 D). These data indicate that the initial antibody-mediated loss of cell adhesion does not require depletion of Dsg3.

### Cell-cell Adhesion is Impaired by Silencing of Dsg3 but not of Dsg2

**Figure 4:**
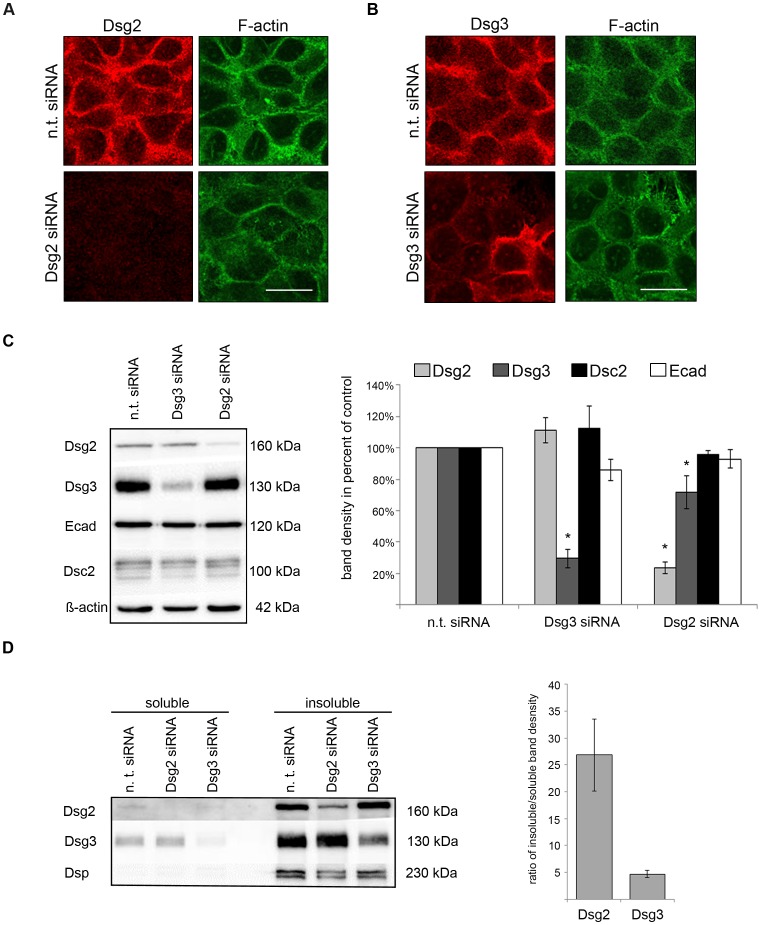
SiRNA-mediated depletion of Dsg2 and Dsg3 in HaCaT cells. (A) Efficient Dsg2 (red) depletion was detected by immunofluorescence staining in HaCaT cells. Actin filaments are colored in green with Alexa Fluor®488 phalloidin. Scale bar, 20 µm. (B) Immunofluorescence staining for Dsg3 (red) and F-actin (green) after siRNA-mediated Dsg3 silencing. Scale bar, 20 µm. (C) Immunoblot analysis demonstrated a decrease in protein expression of either Dsg2 or Dsg3 after respective siRNA-mediated knockdown whereas protein levels of Dsc2 and E-cadherin were not changed after siRNA-mediated silencing of Dsg2 or Dsg3. Dsg2 depletion induced a slight but significant reduction of Dsg3 protein content. In contrast, Dsg2 levels were unchanged after Dsg3 silencing. ß-actin was used as loading control and band density was normalized to ß-actin. (n = 6; * p<0.05 vs. n. t. siRNA) (D) In contrast to Dsg3, endogenous Dsg2 expression was primarily detectable in the desmosomal pool (left panel). SiRNA-mediated gene silencing caused a reduction of Dsg3 in both protein fractions. Desmoplakin was used to identify the Triton X-100-insoluble fraction as the desmosome containing pool. Under control conditions, band density analysis revealed a five times higher ratio of insoluble over soluble protein levels of Dsg2 compared to Dsg3 indicating a more pronounced extradesmosomal localization of Dsg3 (right panel). (n = 5).

The experiments using monoclonal antibodies to interfere with cadherin transinteraction raised the possibility that Dsg2 is dispensable for keratinocyte cohesion when other desmosomal cadherin isoforms are also expressed. However, since the accessibility of antibodies to desmosomes may vary to a certain extent, it can not be completely ruled out that the sensitivity of this approach is limited. Therefore, a siRNA-based approach was used to deplete either Dsg2 or Dsg3 protein levels in HaCaT cells. First, effective knockdown was proven by immunofluorescence and Western blot analysis compared to controls transfected with non-targeting (n. t.) siRNA (Fig. 4 A, B and C). The expression levels of Dsg2 had a minor but significant effect on Dsg3 levels since Dsg2-depleted HaCaT cells (23.6±3.8% of n. t. siRNA controls) showed decreased Dsg3 levels to 71.7±10.4% of n. t. siRNA controls. In contrast, no change in Dsg2 expression (111.4±8.0% of n. t. siRNA controls) was observed after Dsg3 knockdown (29.6±5.9% of n. t. siRNA controls). The protein levels of E-cadherin (Ecad) and Dsc2 were unchanged after either Dsg2 or Dsg3 silencing (Fig. 4 C). Triton X-100 extraction demonstrated that siRNA depletion reduced protein levels of Dsg2 and Dsg3 to a comparable extent in the soluble, i.e. extradesmosomal, as well as in the insoluble, i.e. desmosmal fraction (Fig. 4 D, left panel). Band density analysis revealed a five times higher ratio of insoluble over soluble protein levels of Dsg2 compared to Dsg3 indicating a more pronounced extradesmosomal localization of Dsg3 under control conditions (Fig. 4 D, right panel).

**Figure 5:**
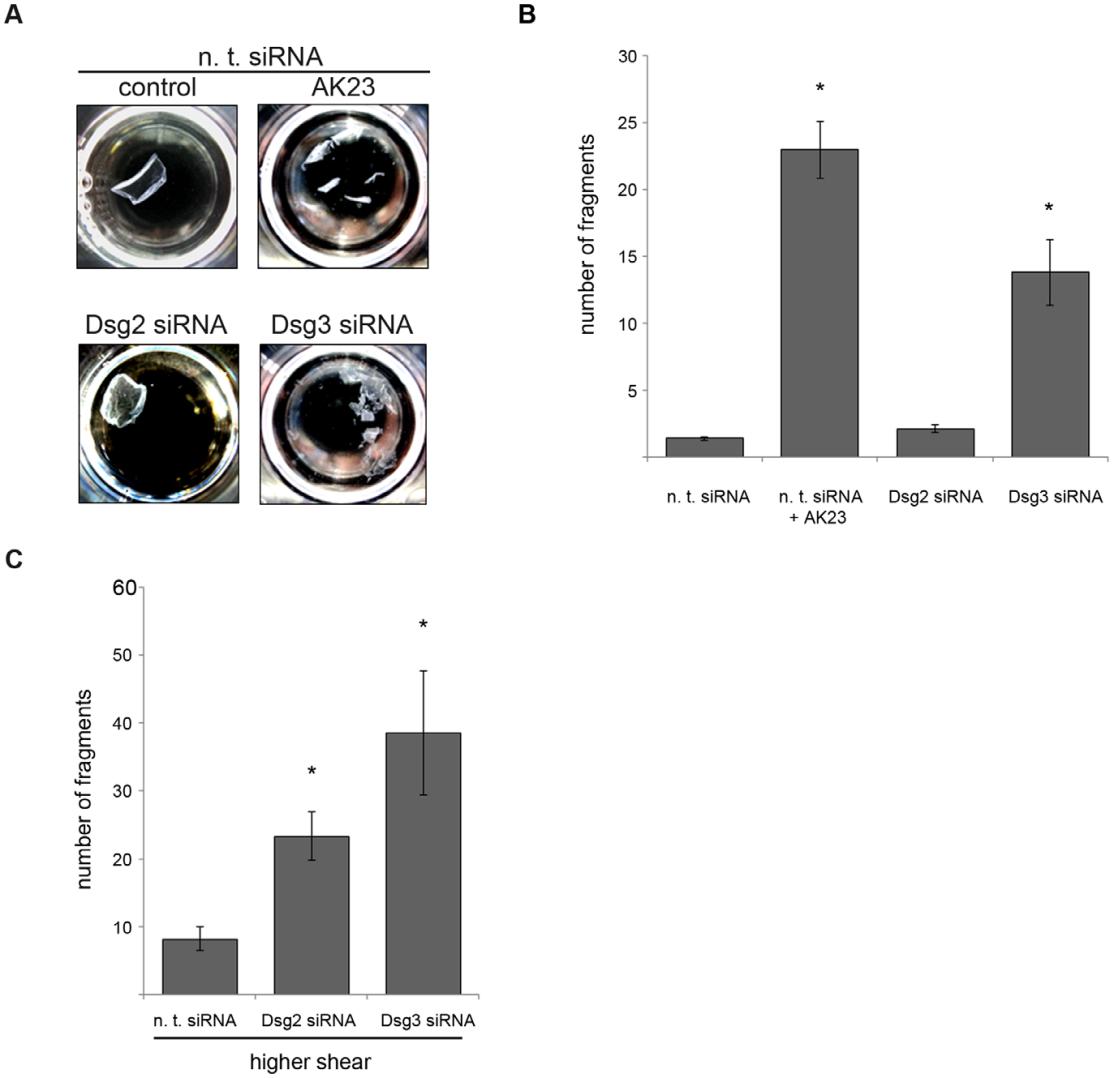
Dsg3 but not Dsg2 depletion leads to loss of cell-cell adhesion. (A) Culture wells photographed after performance of the dispase-based dissociation assay under indicated conditions. Loss of cell-cell adhesion was detectable after AK23 incubation and siRNA-induced Dsg3 knockdown, but absent when Dsg2 levels were reduced by siRNA. (B) Mean fragment numbers per well under experimental conditions when control monolayers withstood the mechanical stress level and stayed intact. (n>20; * p<0.05 vs. n. t. siRNA) (C) Fragment numbers after exposing cell monolayers to higher mechanical stress. (n≥5; * p<0.05 vs. n. t. siRNA).

Under these knockdown conditions the dispase-based assay was used. In accordance with the data using inhibitory antibodies to target desmosomal cadherin function, knockdown of Dsg2 showed no significant increase of fragment numbers (2.08±0.28) compared to cells transfected with n. t. siRNA (1.36±0.1). In contrast, Dsg3-depleted cells were not capable to withstand mechanical stress as revealed by significantly enhanced fragment numbers (13.77±2.44) which were almost comparable to values after incubation with AK23 (22.95±2.11) (Fig. 5 A and B).

To address the possibility that Dsg2 is important for keratinocyte cohesion under high mechanical stress only, experiments were carried out under higher shear. Under these conditions, depletion of both Dsg2 (23.33±3.58) and of Dsg3 (38.58±9.12) significantly increased the number of cell fragments when compared to controls (8.2±1.81) (Fig. 5 C). Taken together, these data indicate that, in contrast to Dsg3, Dsg2 appears to be required for cell cohesion predominantly under conditions when keratinocytes are subjected to higher mechanical forces.

## Discussion

In the present study, we compared the roles of the two desmosomal cadherins Dsg2 and Dsg3 for keratinocyte cohesion, the latter of which is well established to be important for epidermal integrity from its involvement in the pathogenesis of PV [5], in a human cell culture model. Incubation of HaCaT and SCC9 monolayers with monoclonal antibodies targeting Dsg2 (Dsg2 mAb) or Dsg3 (AK23) binding revealed that disrupted Dsg3 transinteraction reduced cell integrity whereas a Dsg2 mAb showed no effect in a dissociation assay. Both monoclonal antibodies were probed by AFM experiments in a cell-free system to confirm their capability to directly interfere with homophilic binding of their target protein. Furthermore, the Dsg2 mAb was also effective to cause cell dissociation in Caco-2 monolayers where Dsg3 is not expressed. Additionally, our data indicate that depletion of Dsg3 is not required for autoantibody-induced loss of keratinocyte cohesion in PV but rather occurred as a subsequent event. We further demonstrated by siRNA-mediated knockdown experiments that Dsg2 in contrast to Dsg3 is less important for keratinocyte adhesion as loss of monolayer integrity was detectable only when Dsg2-depleted cells were subjected to high mechanical shear.

### Dsg2 and Dsg3 Contribute Differently to Keratinocyte Cohesion

Our study supports the crucial role of Dsg3 for keratinocyte cohesion and epidermal integrity which is well established from its loss of function in PV pathogenesis [5]. It was demonstrated previously with the dispase-based dissociation assay that monoclonal antibodies targeting Dsg3 disrupt Dsg3 transinteraction and cause a loss of cell cohesion in cultured keratinocytes as well as in an active pemphigus mouse model [16], [21], [22], [23]. Similarly, genetic deletion of Dsg3 in mice resulted in a phenotype which may resemble PV in its mucosal-dominant form, i.e. when mucous membranes are involved only whereas the epidermis stays intact and patients display autoantibodies against Dsg3 but not Dsg1 [1]. Dsg3-depleted mice suffered from oral erosions and some limited suprabasal epidermal blistering in areas subjected to extensive mechanical stress [27].

In contrast, we were surprised to find that Dsg2, at least under conditions where all other major desmosomal cadherins typical for basal keratinocytes were present, was dispensable for keratinocyte cohesion when low mechanical stress was applied. Both, a monoclonal antibody directed against Dsg2 as well as siRNA-mediated Dsg2 down-regulation did not result in keratinocyte dissociation. We can rule out that the monoclonal antibody did not get access to desmosomes or was not inhibitory under the conditions used because it was effective to reduce homophilic Dsg2 transinteraction as revealed by AFM measurements and similar to a previous study was effective to reduce cohesion of intestinal epithelial cells (Caco-2), underlining an important role of Dsg2 for maintaining intestinal epithelial barrier integrity [9]. These data show that the conditions used for the Dsg2 antibody were appropriate and that Dsg2 in simple epithelia, where Dsg2 and Dsc2 are the only desmosomal cadherins expressed, contributes significantly to intercellular cohesion and barrier function [9].

To reconcile these on first sight conflicting findings it has to be considered that, dependent on the differentiation status, keratinocytes usually express a large set of Dsg and Dsc isoforms that can serve as potential compensation partners for the disrupted intercellular Dsg2 binding, whereas in Caco-2 cells Dsg2 and Dsc2 are the only desmosomal cadherins expressed. Under conditions used for our study, HaCaT cells express Dsg2, Dsg3, Dsc2 (Fig. 4 C) and Dsc3 [16] whereas Dsg1 (and putatively Dsg4 and Dsc1) was found in significant amounts only when cells were further differentiated [1], [14], [28]. In the field of pemphigus research the so-called “desmoglein compensation hypothesis” was established to explain that some Dsg isoforms can compensate for each other: when autoantibodies are present in pemphigus patients targeting both Dsg1 and Dsg3, they may bind as an initial event to the extracellular domain of desmosomal cadherins, disrupt intercellular binding and lead to epidermal blister formation. In contrast, if Dsg1 or Dsg3 is able to compensate for the functional loss of the respective other targeted protein, no blistering occurs in this epidermal layer [29], [30]. However, this hypothesis is controversially discussed and is not likely to be confined to Dsg1 and Dsg3 [28], as for example loss of Dsc3 binding leads to a blistering phenotype similar to loss of Dsg3 binding [31]. Nevertheless, the concept of desmoglein compensation raises the idea that desmosomal cadherins may at least in part be redundant or exchangeable in function. This hypothesis was confirmed by a transgenic mouse model in which increased Dsg2 levels limited superficial blister formation induced by the injection of pemphigus foliaceus (PF) antibodies targeting Dsg1 into neonatal mice [32]. In view of these data, it was unexpected for us to find that targeting Dsg2, an adhesion molecule required for cell cohesion in simple epithelia and able to compensate for Dsg1 loss of function in the epidermis had no effect of cell cohesion in cultured keratinocytes.

This raised the question whether Dsg2 is required for keratinocyte cohesion only under conditions of high mechanical stress. Indeed, when monolayers of Dsg2-depleted cells were subjected to higher shear the number of cell fragments was significantly increased. We conclude that the contribution of specific desmoglein isoforms to overall keratinocyte cohesion may not be similar under all conditions but rather appears to be dependent on the mechanical forces keratinocytes are subjected to. This observation may explain strong expression of Dsg2 in the hair follicle root sheath where mechanical stress can be assumed to be significant.

### Dsg3 Depletion is not Required for Initial Autoantibody-induced Loss of Keratinocyte Cohesion in Pemphigus Vulgaris

Our experiments also give some insight into the pathogenesis of PV. In Pemphigus patients circulating autoantibodies targeting Dsg1 and Dsg3 induce acantholysis within the epidermis and mucous membranes [1]. Different mechanisms for disease pathogenesis are considered. It has been reported that PV-autoantibodies are able to directly impair desmosomal cadherin binding by inhibiting interaction of the Dsg3 binding domains [21], [22], [23]. However, this autoantibody-mediated targeting of adhesion proteins may not cause cell detachment by itself but rather via modifying intracellular signaling pathways [1], [33]. A variety of signaling mechanisms has been reported to be altered upon autoantibody binding including p38MAPK activation [34], [35], [36], [37], PKC activation [38], cAMP signaling [39] and RhoA inhibition [40], [41], among others. These events, which at least in part appear to be dependent on autoantibody-induced loss of Dsg3 function impair desmosomal adhesion and finally lead to loss of cell-cell cohesion in pemphigus. In this context it was proposed that depletion of Dsg3 via clathrin-independent endocytosis [42], which may also involve p38MAPK [36], is a central mechanism because exogenous overexpression of Dsg3 blocked autoantibody-induced loss of cell cohesion [43]. Our results do not contradict these findings but indicate that the initial loss of both Dsg3 binding, as revealed by laser tweezer trapping of Dsg3-coated microbeads, and keratinocyte cohesion, as measured by the dispase-based assay, does not require Dsg3 depletion because both events were detectable as soon as after 30 min whereas Dsg3 depletion in the extradesmosomal fraction was not observed within the first 6 h, at least under the conditions used for this study. Rather, Dsg3 depletion appears to take place after initial loss of Dsg3 binding and may further aggravate loss of cell cohesion. In line with this, it was proposed recently that PKC-mediated Dsg3 depletion [44] which was found to be most prominent in basal keratinocytes, in addition to other mechanisms may trigger blister formation in the lower epidermis where it is typical for the histology of PV lesions [38].

### Possible Function of Dsg2 Besides Serving as a Molecule Important for Cell Adhesion

We were unable to detect Dsg2 in intact epidermis outside of hair follicles fitting to the idea that Dsg2 is not crucial for epidermal integrity and overall keratinocyte cohesion. Nevertheless, strong expression in keratinocytes of the hair follicle root sheath indicates that Dsg2 supports keratinocyte cohesion under high mechanical stress. In this localization, Dsg2 levels were reported to be regulated by matriptase [45].

Indeed, Dsg2 may be involved in intracellular signaling events. In skin samples of transgenic mice expressing Dsg2 under control of the involucrin promoter, activation of several pathways such as PI3-kinase/AKT, MEK-MAPK, STAT3 and NF-kB was detectable and these mice were more vulnerable to chemically induced carcinogenesis [46]. Moreover, Dsg2 was shown recently to interact with caveolin-1 and proposed to be important for desmosome turnover [10]. In line with this, it was found to be up-regulated in skin-derived carcinomas and therefore is discussed to serve as cancer marker [19]. Others claim a role of Dsg2 for regulating apoptosis in the intestinal epithelium, as siRNA-mediated depletion of Dsg2 was protective against induction of apoptosis [26]. Thus, it is conceivable that desmogleins serve as initiators and modulators of signaling events in the epidermis. This is underscored by recent data reporting Dsg3 to regulate Src in cooperation with E-cadherin [3] as well as actin cytoskeleton reorganization via Rho family GTPases [47]. However, no such data are available for Dsg2. Unfortunately, to examine the effects of Dsg2 gene silencing *in vivo* no animal model is available so far as Dsg2 knockout mice exhibit early embryonic lethality [48]. Nevertheless, further studies are necessary to elucidate the signaling functions of Dsg2 in keratinocytes.

## Supporting Information

Figure S1 **Expression profile of desmosomal cadherins in 4 day HaCaT cells.** (A) Immunofluorescence staining of Dsg1-3 and Dsc1-3 in HaCaT cells. Scale bar, 20 µm. (B) Immunoblot detection of Dsg1 in 4 day and 8 day HaCaT cells demonstrated this protein to be expressed in relevant amounts after 8d only. (n = 2)
(TIF)

Figure S2 **Targeting of Dsg2 and Dsg3 function in SCC9 and HT-29 cells.** (A) Immunofluorescence staining of Dsg2 and Dsg3 in 5d SCC9 cells. Scale bar, 20 µm. (B) Expression profile of desmosomal cadherins in 5d HaCaT and 5d SCC9 cells. (C) A significant loss of SCC9 cell cohesion was detectable after 24 h incubation with AK23 but not with Dsg2 mAb in dissociation assays. (n>11; * p<0.05 vs. control) (D) siRNA-mediated depletion of Dsg2 in adenocarcinoma cells (HT-29) reduced cell cohesion in the dispase-based dissociation assay. (n = 6; * p<0.05 vs. n. t. siRNA) (E) Successful knockdown of Dsg2 in HT-29 cells proven by Western blot analysis. α-Tubulin was used as loading control. (n = 3)
(TIF)

Figure S3 **Antibody-targeting of Dsg2 and Dsg3 does not block desmosomal reconstitution in Ca^2+^-switch assays.** Both Dsg2 mAb and AK23 did not block the distribution of Dsg2 (red, upper panel) and Dsg3 (red, lower panel) to nascent junctions 18 h after increasing Ca^2+^-levels in HaCaT cells. Staining for actin filaments (F-actin; green) served to delineate intercellular gap formation.
(TIF)

Figure S4 **Dsg2 mAb and AK23 are both detectable after 24 h incubation on HaCaT cells.** (A) Binding of Dsg2 mAb as well as of AK23 to HaCaT cells was demonstrated in the desmosomal (Triton X-100-insoluble) fraction by delineating the heavy and light chains using a mouse HRP-conjugated secondary antibody. (n = 3)
(TIF)
