# Supplementary figures and images for: Desmoglein 2 Is Less Important than Desmoglein 3 for Keratinocyte Cohesion
Source: PLoS One. 2013 Jan 11;8(1):e53739. doi: 10.1371/journal.pone.0053739 (PMC3543261; doi:10.1371/journal.pone.0053739)

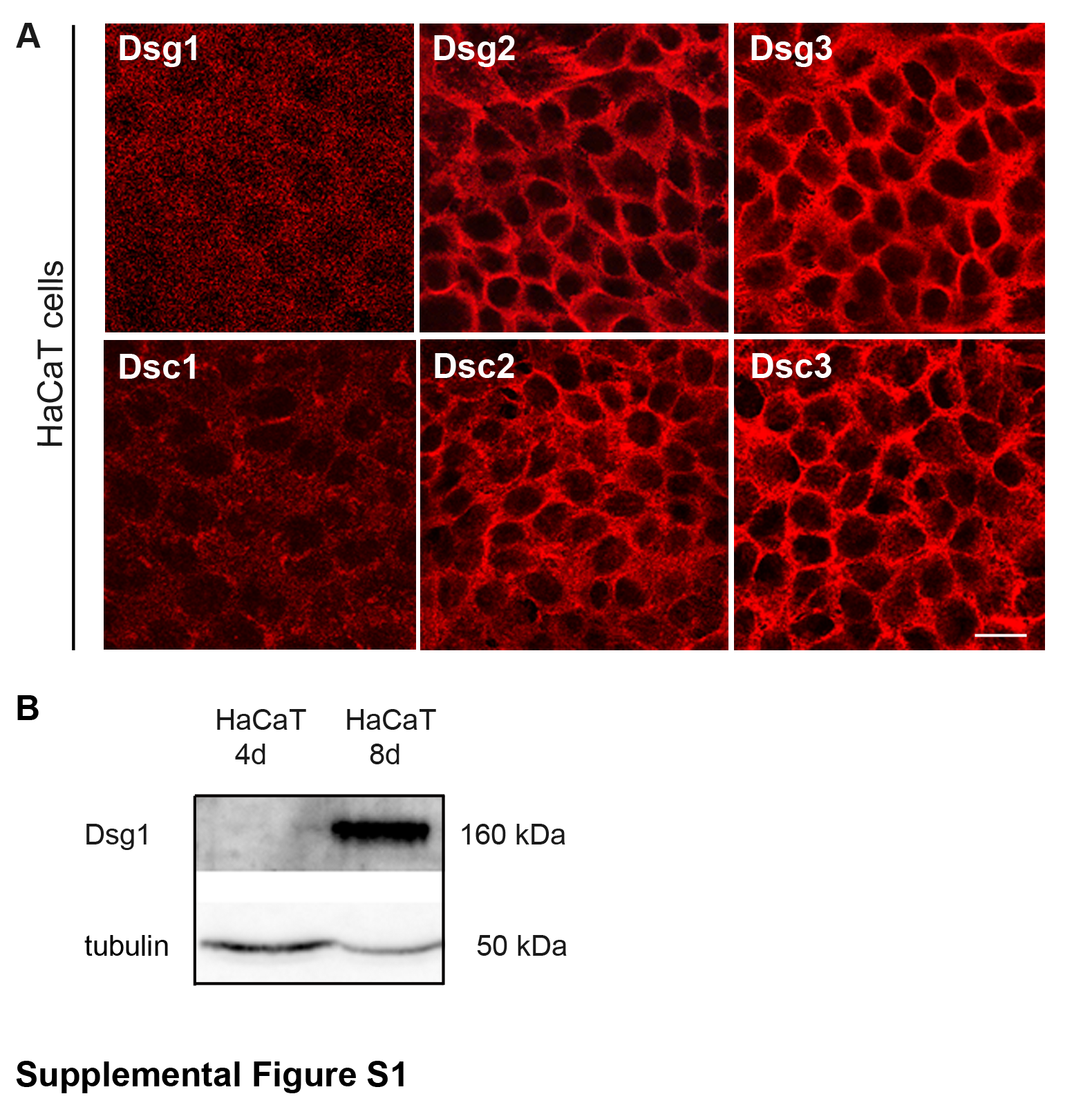

Supplement: Figure S1 — Expression profile of desmosomal cadherins in 4 day HaCaT cells. (A) Immunofluorescence staining of Dsg1-3 and Dsc1-3 in HaCaT cells. Scale bar, 20 µm. (B) Immunoblot detection of Dsg1 in 4 day and 8 day HaCaT cells demonstrated this protein to be expressed in relevant amounts after 8d only. (n = 2) (TIF) [file pone.0053739.s001.tif]

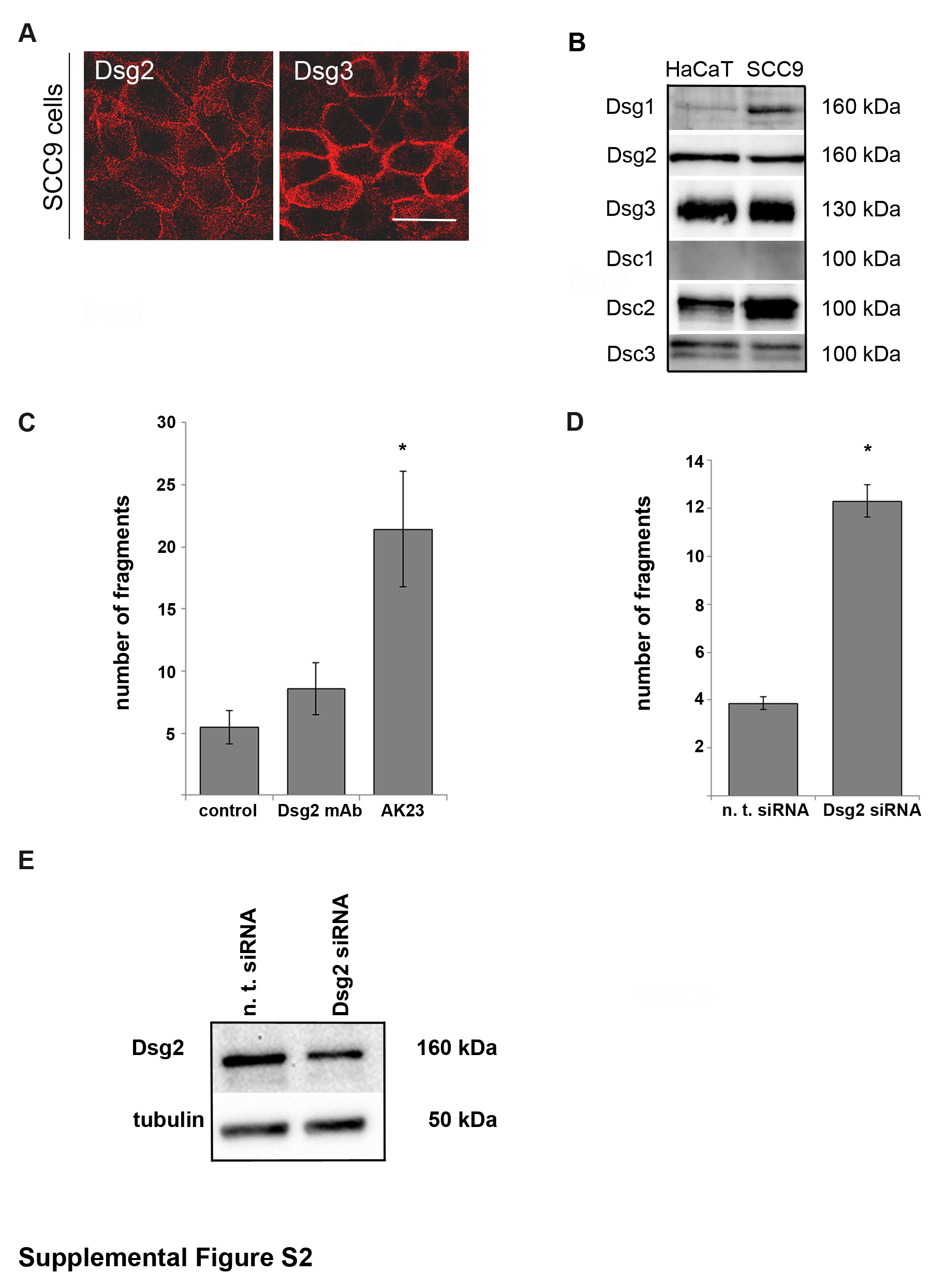

Supplement: Figure S2 — Targeting of Dsg2 and Dsg3 function in SCC9 and HT-29 cells. (A) Immunofluorescence staining of Dsg2 and Dsg3 in 5d SCC9 cells. Scale bar, 20 µm. (B) Expression profile of desmosomal cadherins in 5d HaCaT and 5d SCC9 cells. (C) A significant loss of SCC9 cell cohesion was detectable after 24 h incubation with AK23 but not with Dsg2 mAb in dissociation assays. (n>11; * p<0.05 vs. control) (D) siRNA-mediated depletion of Dsg2 in adenocarcinoma cells (HT-29) reduced cell cohesion in the dispase-based dissociation assay. (n = 6; * p<0.05 vs. n. t. siRNA) (E) Successful knockdown of Dsg2 in HT-29 cells proven by Western blot analysis. α-Tubulin was used as loading control. (n = 3) (TIF) [file pone.0053739.s002.tif]

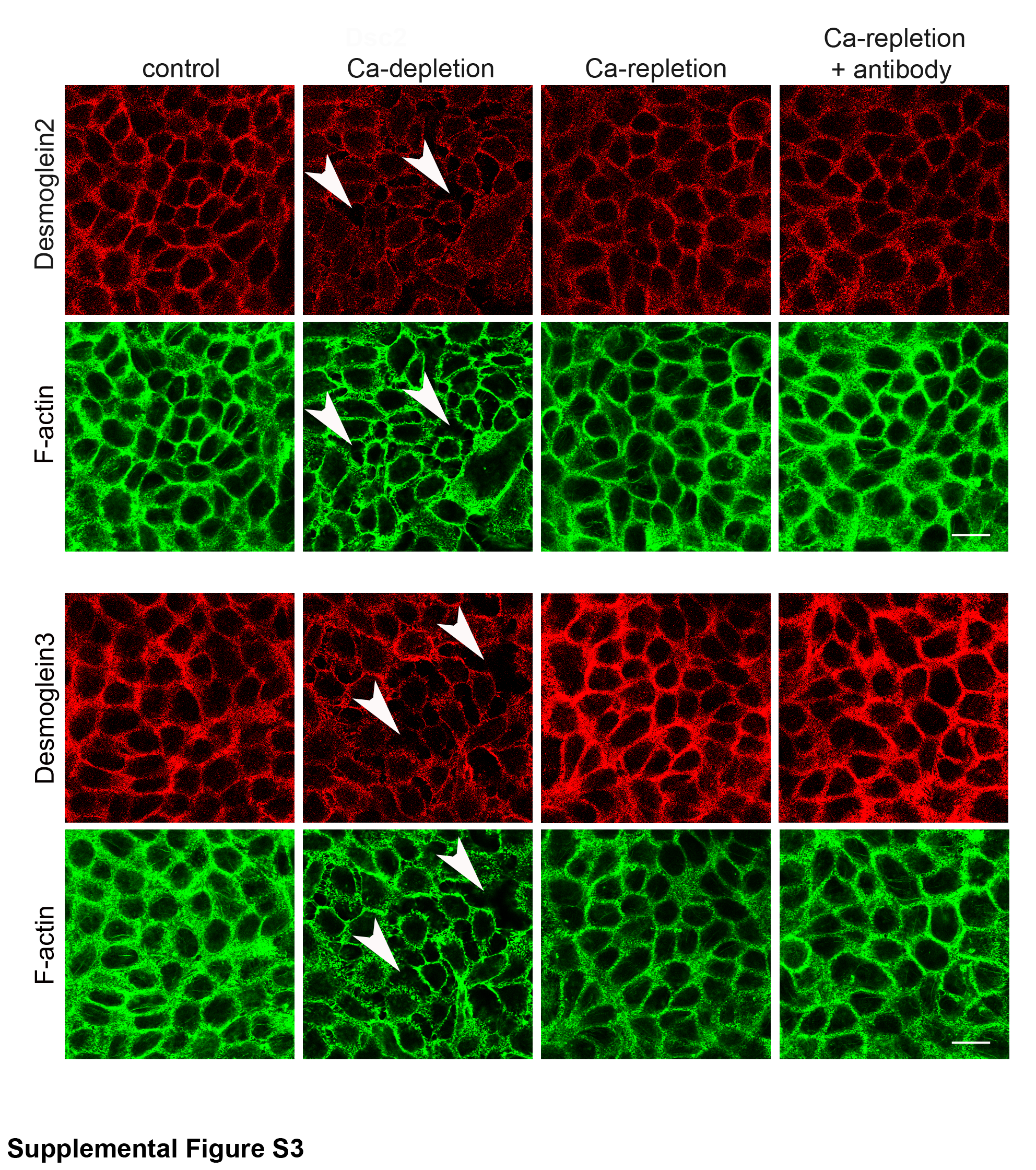

Supplement: Figure S3 — Antibody-targeting of Dsg2 and Dsg3 does not block desmosomal reconstitution in Ca2+-switch assays. Both Dsg2 mAb and AK23 did not block the distribution of Dsg2 (red, upper panel) and Dsg3 (red, lower panel) to nascent junctions 18 h after increasing Ca2+-levels in HaCaT cells. Staining for actin filaments (F-actin; green) served to delineate intercellular gap formation. (TIF) [file pone.0053739.s003.tif]

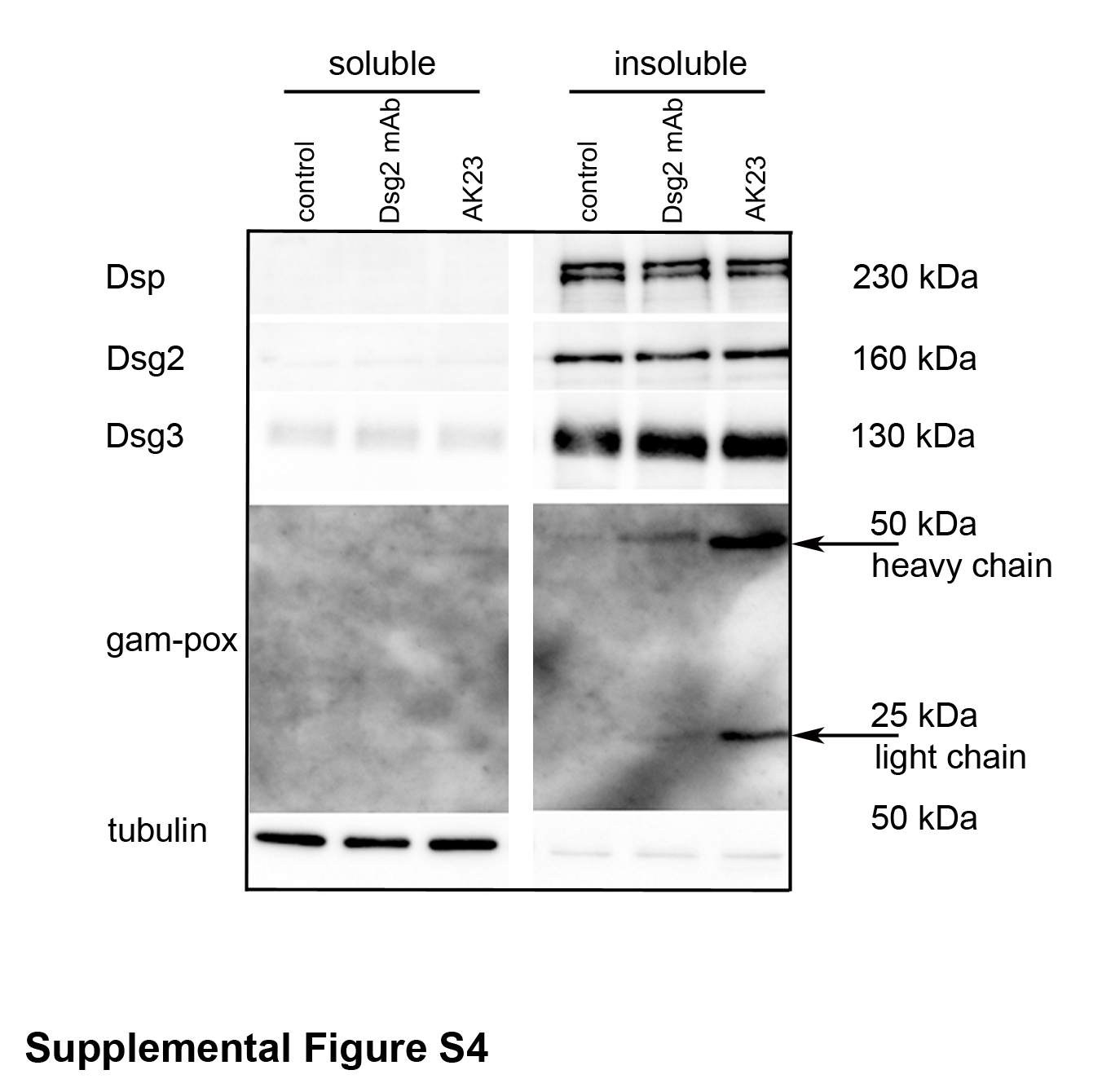

Supplement: Figure S4 — Dsg2 mAb and AK23 are both detectable after 24 h incubation on HaCaT cells. (A) Binding of Dsg2 mAb as well as of AK23 to HaCaT cells was demonstrated in the desmosomal (Triton X-100-insoluble) fraction by delineating the heavy and light chains using a mouse HRP-conjugated secondary antibody. (n = 3) (TIF) [file pone.0053739.s004.tif]
